# Combined B-type Natriuretic Peptide as strong predictor of short-term mortality in patients after Liver Transplantation

**DOI:** 10.7150/ijms.54202

**Published:** 2021-04-23

**Authors:** Hyun Sik Chung, AMi Woo, Min Suk Chae, Sang Hyun Hong, Chul Soo Park, Jong Ho Choi, Yun Sung Jo

**Affiliations:** 1 Department of Anesthesiology and Pain Medicine, Eunpyeong St. Mary's Hospital, College of Medicine, The Catholic University of Korea, Seoul, Republic of Korea.; 2 Department of Anesthesiology and Pain Medicine, Seoul St. Mary's Hospital, College of Medicine, The Catholic University of Korea, Seoul, Republic of Korea.; 3 Department of Obstetrics and Gynecology, St. Vincent's Hospital, College of Medicine, The Catholic University of Korea, Gyeonggi, Republic of Korea.

**Keywords:** B-type natriuretic peptide, liver transplantation, mortality, prognosis, sensitivity, specificity

## Abstract

**Background:** B-type natriuretic peptide (BNP) is a well-known predictor for prognosis in patients with cardiac and renal diseases. However, there is a lack of studies in patients with advanced hepatic disease, especially patients who underwent liver transplantation (LT). We evaluated whether BNP could predict the prognosis of patients who underwent LT.

**Material and Methods:** The data from a total of 187 patients who underwent LT were collected retrospectively. The serum levels of BNP were acquired at four time points, the pre-anhepatic (T1), anhepatic (T2), and neohepatic phases (T3), and on postoperative day 1 (T4). The patients were dichotomized into survival and non-survival groups for 1-month mortality after LT. Combined BNP (cBNP) was calculated based on conditional logistic regression analysis of pairwise serum BNP measurements at two time points, T2 and T4. The area under the receiver operating characteristic curve (AUROC) was analyzed to determine the diagnostic accuracy and cut-off value of the predictive models, including cBNP.

**Results:** Fourteen patients (7.5 %) expired within one month after LT. The leading cause of death was sepsis (N = 9, 64.3 %). The MELD and MELD-Na scores had an acceptable predictive ability for 1-month mortality (AUROC = 0.714, and 0.690, respectively). The BNPs at each time point (T1 - T4) showed excellent predictive ability (AUROC = 0.864, 0.962, 0.913, and 0.963, respectively). The cBNP value had an outstanding predictive ability for 1-month mortality after LT (AUROC = 0.976). The optimal cutoff values for cBNP at T2 and T4 were 137 and 187, respectively.

**Conclusions:** The cBNP model showed the improved predictive ability for mortality within 1-month of LT. It could help clinicians stratify mortality risk and be a useful biomarker in patients undergoing LT.

## Introduction

Several established scoring systems, including a model for Child-Turcotte-Pugh (CTP), end-stage liver disease (MELD), and sequential organ failure assessment (SOFA), have been introduced to estimate the mortality in patients undergoing liver transplantation (LT) 1-3. With respect to the MELD score, the pretransplant MELD score is also a well-known predictive model for estimating the early mortality of patients undergoing LT 4. Other than these scoring systems, some biomarkers, such as proteinuria have been used to estimate post-LT mortality 5. With respect to cardiac markers, previous studies have been reported a correlation between serum troponin levels and cardiovascular events or graft survival after LT 6, 7. However, B-type natriuretic peptide (BNP) has been poorly studied in this context, although its ability to predict mortality in cardiac or septic patients has been investigated 8-10. Recent studies on the usefulness of serum BNP levels in patients with advanced liver disease reported that the postoperative BNP level was a risk factor predicting deteriorated outcomes after LT, with a suggested postoperative BNP cutoff value of above 400 pg/mL 11.

BNP is a neurohormone that is secreted mainly from the cardiac ventricles in response to tension of the cardiac wall caused by volume and pressure 12, 13. Therefore, increased serum BNP levels in patients with cardiac dysfunction have attracted interest to both the diagnostic and prognostic properties of BNP 14. Serial BNP monitoring is used as a screening test in the intensive care unit to evaluate patients who may develop heart failure and need further evaluation, such as echocardiography, to evaluate the causes of heart failure. In this context, the prognostic ability of BNP levels is worth evaluating in patients undergoing LT, because cardiac death is the leading cause of mortality following LT 15.

In the present study, we evaluated whether BNP had the clinical significance and prognostic ability to predict patient prognosis. We also suggested a new predictive model using BNP by logistic regression analysis of mortality within 1-month of LT, and compared it to pretransplant MELD and MELD-Na scores, which were known to predict mortality after LT.

## Material and Methods

Patients who underwent LT between March 2016 and November 2019 at Seoul St. Mary's Hospital were included in the study. A total of 204 adult patients (≥ 19 years old) were enrolled in the study. The data were collected retrospectively from our hospital's electronic medical record system. The Institutional Review Board approved the use of the registry data for the patients in our hospital. Owing to the retrospective nature, the requirement for written informed consent to use the data for publication was waived by the Institutional Review Board (KC19RESI0204).

The LT surgical procedures were conducted according to the LT protocol of Seoul St. Mary's Hospital. LT was performed by a piggyback technique using the right hepatic lobes of the donor in living donor LT, and replacing the recipient's retrohepatic vena cava with a donor vena caval segment using end-to-end anastomosis between the graft IVC and the recipient IVC in deceased donor LT. In each case, portal vein and hepatic vein anastomoses were performed first, followed by hepatic artery anastomosis and bile duct reconstruction. A venovenous bypass with a pump was not performed. A temporary surgical portocaval shunt during the anhepatic period to decompress the splanchnic circulation and reduce bowel edema was placed in limited patients with minimal collateral circulation. This was based on preoperative computed tomography findings or a high-pressure gradient (> 5 mmHg) between the portal venous pressure after placement of a catheter at the portal vein and the central venous pressure in the central venous catheter at the internal jugular vein in the perioperative period caused by complete clamping of the portal vein. Histidine-tryptophan-ketoglutarate solution (Custodiol^®^ HTK; Dr. Franz Köhler Chemie GmbH, Bensheim, Germany) was used to preserve the graft livers.

The intraoperative anesthetic management followed our institute's protocol for LT. For anesthesia, balanced anesthesia was provided using 1.5-2% sevoflurane or 4-6% desflurane with remifentanil infusion at a rate of 0.1-0.2 μg/kg/min under guidance by bispectral brain monitoring. Atracurium was continuously administrated at a rate of 6-8 μg/kg/min for muscle relaxation. After the induction of anesthesia, a Swan-Ganz catheter was inserted into the right internal jugular vein to provide continuous hemodynamic monitoring, including stroke volume, cardiac output, and vascular resistance measurements. For frequent blood sampling and continuous blood pressure monitoring, the radial artery was cannulated and a 22-gauge angiocatheter was placed. The patient hematocrit was maintained between 25 and 30%, and serum calcium levels and pH were maintained at 80% of the lower limit of the normal range (0.9 mmol/L) of serum ionized calcium 16 and > pH 7.15, respectively, by administering calcium gluconate and sodium bicarbonate with adequate ventilation.

Serial laboratory tests were routinely performed during surgical the phases of LT, which were during the intraoperative period, 60 mins into the pre-anhepatic phase, 30 mins into the anhepatic phase, and 30 mins into the neohepatic phases. Each set of laboratory test included to complete blood count with a differential blood count, blood chemistry with arterial and venous blood gas analysis, tests for disseminated intravascular coagulation, BNP, blood viscosity, and thromboelastogram. Additional laboratory tests were performed at the clinician's discretion. BNP was routinely measured on postoperative day 1 as well as at four time points during the intraoperative period according to the surgical phases (60 mins into the pre-anhepatic phase (T1), 30 mins into the anhepatic phase (T2), and 30 mins after reperfusion of the grafted liver (neohepatic phase, T3)) and on postoperative day 1 (T4). Postoperative BNP was routinely measured at postoperative day 1 and additional BNP measurements were performed if the patients had a higher BNP level with simultaneous cardiac diseases. Each BNP sample was collected using the nearest time point within 10 mins of the study time point to minimize confounding by time. The MELD score and MELD-Na score were calculated using previously published formulas 17, 18, and higher MELD and MELD-Na scores indicated more severe hepatic disease.

The primary endpoint was 1-month mortality after LT defined as all-cause mortality during hospitalization. The patients were dichotomized into two groups, survival and non-survival groups.

The variables potentially related to 1-month mortality (*P* < 0.10) were selected using the results from univariate analysis. For calculation of a better prognostic model, multivariate analysis was done. Conditional logistic regression analysis was performed for pairwise interacting variables of two significant BNP time points to create a new prognostic model by combining the BNPs of the T2 and T4, which was called the combined-BNP (cBNP). The BNPs were logarithmically transformed to normal distribution to apply logistic regression. We called the model the combined-BNP (cBNP) model because the model was calculated using two significantly different time points for the BNPs, T2 and T4. The new predictive model was calculated by:

cBNP = 2.56 × Ln BNP (anhepatic phase (T2), pg/mL) + 2.48 × Ln BNP (post-operative day 1 (T1), pg/mL) - 28

To evaluate the fitness of the cBNP model, the Hosmer-Lemeshow goodness of fit test for logistic regression was performed.

The individual diagnostic accuracy of cBNP, BNP, Cr (creatinine), MELD scores, and MELD-Na scores for mortality within 1-month after LT was investigated using the area under the receiver operator characteristic curve (AUROC), and the threshold scores, sensitivities, specificities, positive predictive values, and negative predictive values were calculated. We calculated the discrimination of individual AUROCs using the improvement in individual AUROC models by calculating the difference in the AUROCs (ΔAUROC). The AUROCs were compared by a method proposed by DeLong *et al.* 19.

We calculated the net reclassification improvement (NRI) and integrated discrimination improvement (IDI) to quantify the incremental predictive value between the cBNP and MELD scores from the AUROC analysis resulting in reclassification. The NRI and IDI were calculated by a formula proposed by Pencina *et al*. 20. We classified the predicted risk into four strata (0-5, 5-15, 15-20, and > 20% 1-month mortality risk) for mortality within 1-month after LT.

Statistical analyses were performed using R software version 4.0.2 (R Foundation for Statistical Computing, Vienna, Austria). The study population data are presented as means ± standard deviations (SD), medians (interquartile ranges, IQR), or absolute values (proportions) as appropriate. All variables were analyzed using parametric or nonparametric tests as appropriate, followed by the Kolmogorv-Smirnov test to determine the normal distribution of each value. Student's t-tests, or Mann-Whitney test (for continuous variables) and Chi-squared tests, or Fisher's exact test (for categorical variables) were performed to compare the two groups. Conditional logistic regression was done to calculate a new model and the Hosmer-Lemeshow goodness of fit test was performed. The diagnostic accuracy and cutoff value of the models were analyzed using the AUROC. All *P* values were two-sided, and a *P* value of < 0.05 was considered statistically significant.

## Results

**Figure 1:**
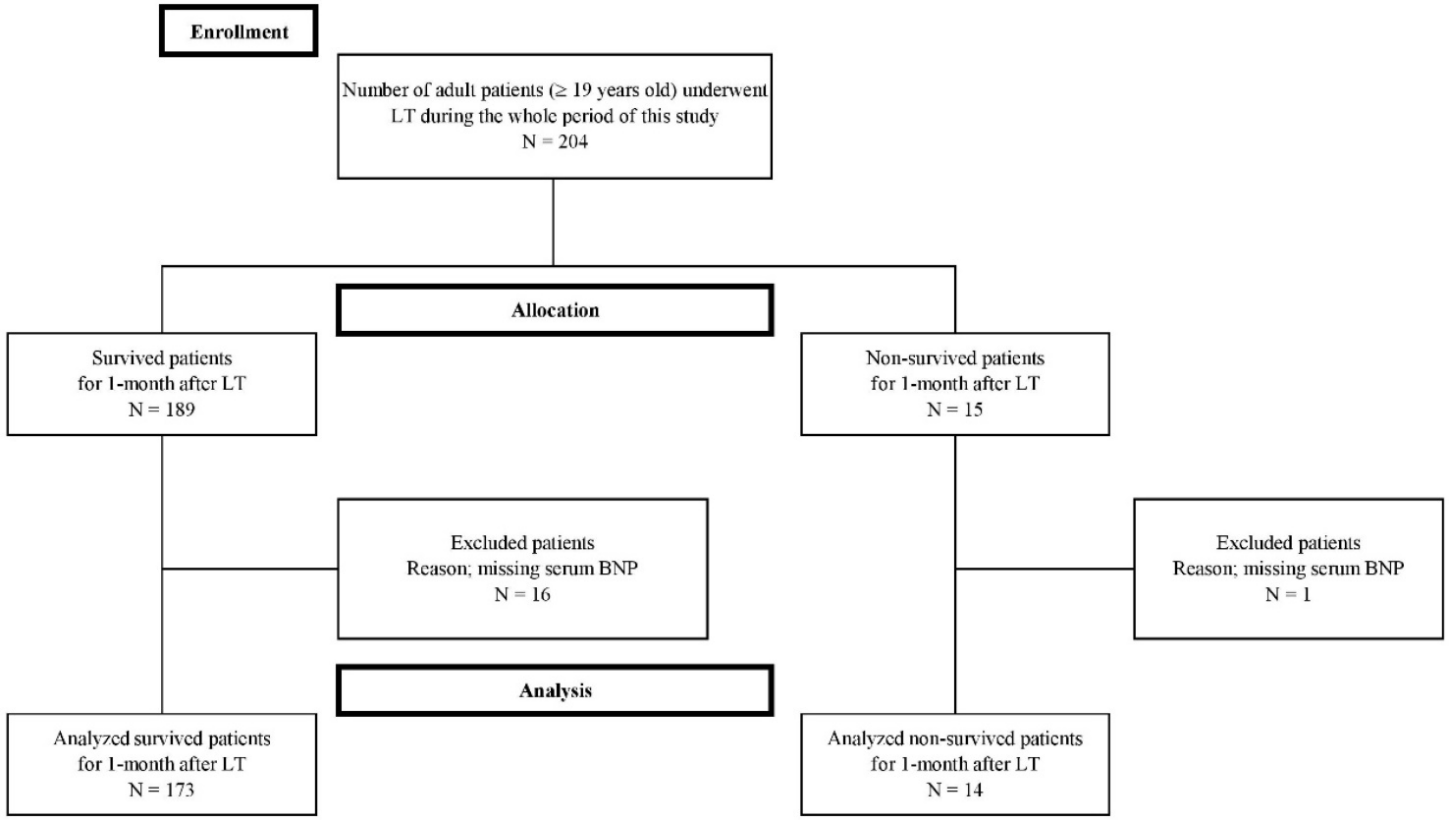
Flow diagram of the study.

Seventeen cases were excluded from this study because they were missing laboratory BNP data needed in the study protocol. Finally, a total of 187 patients were enrolled in the present study (Figure 1). The enrolled patients were dichotomized into two groups in terms of mortality within 1-month after LT, the survival and non-survival groups. Fourteen (7.5 %) of 187 patients expired within one month after LT and 173 patients survived. The most common cause of mortality was sepsis (N = 9, 64.3%), followed by vascular complication (N = 3, 21.4%), and graft failure (N = 2, 14%). Of the patients with sepsis, the most common manifestation was pneumonia (N = 6, 66.7%), followed by biliary sepsis (N = 2, 22.2%), and unknown origin (N = 1, 11.1%). The vascular complications included hepatic artery thrombosis (N = 2, 66.7%), and hepatic vein stenosis (N = 1, 33.3%). Five patients (35.7%) of 14 non-surviving patients expired within the first seven days after LT, five (35.7%) of 14 non-surviving patients expired between 8 and 21 days after LT, and four (28.6%) of 14 non-surviving patients expired from 22 days to 30 days after LT.

The demographic and preoperative data of the recipients and donors are shown in Table 1. There were more male patients than female patients in both the survival and non-survival groups (68.8% males in the survival group vs. 64.3% in the non-survival group). However, there were no significant differences between the two groups. In the scoring system and preoperative laboratory data, the non-survival group had higher MELD scores, MELD-Na scores, international normalized ratios (INR), Cr, and C-reactive protein than the non-survival group (*P* < 0.05). With respect to the causes of end-stage liver disease (ESLD), there was a significant difference between the two groups (*P* < 0.05). The survival group showed a higher proportion of viral-associated ESLD than non-viral ESLD (viral ESLD, 56.6%; non-viral ESLD, 43.4%). However, the difference between viral and non-viral ESLD in the non-survival group was not significantly different (viral ESLD, 50.0%; non-viral ESLD, 50.0%). Among the viral-associated ESLD, hepatitis B virus infection was the predominant cause in the survival group (40.5%), and hepatitis A virus and hepatocellular carcinoma were the predominant causes in the non-survival group (21.4% and 21.4%; respectively). With respect to preoperative echocardiography, the ejection fraction (EF) and systolic dysfunction (EF < 50 %) were not significantly different between the two groups. However, there was a higher proportion of patients with systolic dysfunction (EF < 50 %) in the non-survival group than in the survival group (*P* = 0.220). The proportion of patients with diastolic dysfunction and pulmonary hypertension was also not significantly different between the two groups. There were no statistically significant differences between the two groups in demographics and preoperative laboratory data of the recipients and the donors, including age, body mass index, neutrophil-to-lymphocyte (NL) ratio, serum electrolytes, ischemic time of the liver graft, the graft-to-recipient weight ratio (GWRW), and fatty changes in the grafted livers (Table 1).

Table 2 shows the perioperative data, including laboratory and hemodynamic parameters according to the three surgical LT phases, pre-anhepatic (T1), anhepatic (T2), and neohepatic phases (T3), and the BNP and neutrophil-to-lymphocyte ratio on postoperative day 1 (T4). The intraoperative and postoperative BNP levels were significantly different between the survival and the non-survival groups in all phases. The BNP levels were higher in the non-survival group than the survival group. The pH was significantly lower only in the pre-anhepatic phase and the CVP was higher level in the non-survival group than the survival group only in the neohepatic phase (*P* < 0.05). However, the pH and CVP levels showing a statistical significance between the two groups were within the normal ranges. The creatinine levels, administered fluids, number of patients with ascites > 1L, and operation time were not significantly different between the two groups, and mean arterial blood pressure (mABP), stroke volume variation (SVV), cardiac index (CI), and systemic vascular resistance index (SVRI) were also not significantly different between the two groups in the perioperative period.

The BNP values were log-transformed due to the skewness of the BNP value distribution and multivariate adjustment was done using the results from the univariate analysis of the BNPs in logistic regression. Table 3 shows that the 1-month mortality increased 12.9 and 12.0 times when the LN BNPs at T2 and T4 increased by one point, respectively. We calculated the cBNP using the results from conditional logistic regression and evaluated the goodness of fit of the regression model using the Hosmer and Lemeshow test. The Hosmer and Lemeshow test showed a good fit of the cBNP model with a χ^2^ and *P* value of 1.24 and 0.996, respectively 21.

**Figure 2:**
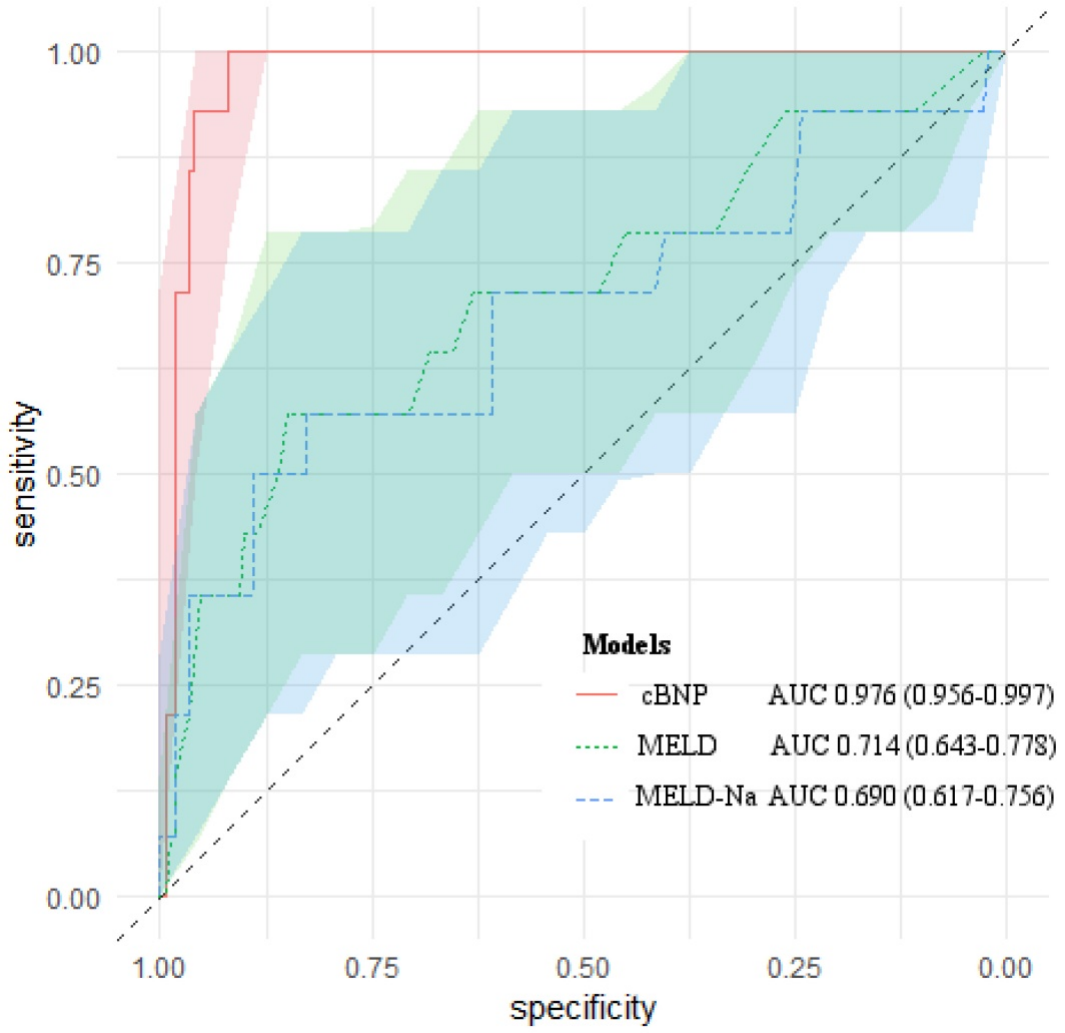
AUROCs of cBNP, MELD scores and MELD-Na scores for predicting 1-month mortality after liver transplantation. The light-colored areas indicate 95 % confidence regions for the AUROCs.

We compared the predictive ability of cBNP, each BNP time point, the MELD scores, the MELD-Na scores for 1-month mortality using the AUROC (Table 4). All of them had statistical significance in predicting 1-month mortality after LT (*P* < 0.05). The cBNP was significantly different from the MELD scores, and the MELD-Na scores from the AUROC analyses (ΔAUROC 0.262, and 0.286,* P* = 0.001, and *P* < 0.001, respectively). The cBNP showed the highest predictive accuracy, and the BNP value at postoperative day 1 (T4) had the next best accuracy predicting 1-month mortality (95% CI: 0.941-0.992; AUROC = 0.976 and 95% CI: 0.925-0.985; AUROC = 0.963, respectively). The most discriminatory cutoff values for short-term mortality within 1-month after LT determined using AUROC analyses were an LN BNP level at T2 of > 4.942 and an LN BNP at T4 of > 5.228 for the cBNP, and a BNP at T2 of > 175 pg/mL, a BNP at T4 of > 155 pg/mL, a MELD score > 31, and a MELD-Na score > 29. LN BNP values of 4.942 and 5.228 were equivalent to BNP values of 137 pg/mL, and 187 pg/mL, respectively. At this cutoff point, the cBNP and MELD scores showed 100% and 57.1% sensitivity, and 91.9% and 85.9% specificity, respectively. Figure 2 shows AUROCs and 95% CIs for the cBNP, MELD scores, and MELD-Na scores.

Table 5 summarizes the results from the reclassification of the individual models using the cBNP values and MELD scores. Eleven individuals who survived for 1-month after LT were reclassified up and 63 individuals were reclassified down. It improved the net gain with a reclassification proportion of 0.301. Of the patients who did not survive one month after LT, eight were reclassified up and two were reclassified down. It improved the net gain in the reclassification proportion to 0.429. Therefore, the NRI was estimated at 0.301 (95%CI: 0.224-0.396) and was significantly different (*P* = 0.004). The IDI was estimated at 0.005 (95%CI: 0.027-0.076) and was also significant (*P* = 0.035).

## Discussion

We proposed cBNP as a new predictive BNP model that showed better diagnostic accuracy than the other models using BNP levels at single time points, the MELD score, or the MELD-Na score to predict mortality within 1-month for all causes after LT. Our results showed that the BNP level at a single time point was also suitable for predicting the mortality in patients who underwent LT. However, the combination of BNP levels from two different time points showed improved predictive accuracy for 1-month mortality in patients who underwent LT compared to BNP values at single time points. Moreover, cBNP had better predictive value than MELD scores, even if the MELD score was created to predict the severity of hepatic disease in patients with ESLD 22-24.

Several studies have reported predictive models for mortality after LT, including pretransplant MELD scores, and MELD-Na scores 4, 25-28. The MELD scores well-known useful predictors of early mortality after LT, with higher MELD scores associated with higher mortality, although the MELD score was designed to estimate the survival in patients with elective placement of a trans-jugular intrahepatic portosystemic shunt for portal hypertension 29, 30. Therefore, we tried to compare the serum BNP levels and a new predictive model to the MELD score, which is a representative predictor related to the mortality in patients undergoing LT, to confirm the predictive ability of the suggested a new model.

BNP is secreted from the ventricular muscle of the heart into the circulation in response to tension stress on the cardiac wall and has a half-life in the circulation of about 20 mins 31-33. BNP levels have been used to predict the mortality and morbidity of patients with cardiac diseases, with higher BNP levels correlating with greater risk 34-36. A cutoff value above 100 pg/ml BNP was recommended for a diagnosis of heart failure 37, 38. However, the usefulness of BNP levels related to mortality after LT in patients with ESLD has been poorly evaluated. Two studies have been conducted on the relationship between BNP and the patients with liver disease. One reported that BNP was related to the severity of liver cirrhosis in non-alcoholic patients. The study demonstrated that the patients with higher BNP levels showed poor Child-Pugh classification and more advanced cirrhosis 39. The other evaluated whether pretransplant and posttransplant BNP could predict mortality after LT 11. The study reported that posttransplant BNP was associated with mortality and poor outcomes after LT. The suggested cutoff value of BNP on posttranplant day 3 was > 400 pg/mL. We evaluated the usefulness of BNP to predict mortality after LT using MELD scores instead of the Child-Pugh classification, and BNP levels in intraoperative period and on postoperative day 1. Our results showed that values above 120 to 190 pg/mL were cutoff values for perioperative and postoperative day 1 BNP levels, for the diagnosis of mortality within one month after LT.

Several studies have reported elevated BNP levels in patients with ESLD. Elevated BNP levels in patients with cirrhotic liver disease were used to explained cirrhotic cardiomyopathy and silent heart failure. Some patients showed elevated BNP levels in patients with EFs within the normal range 40 because BNP is a marker of early-stage heart disease. Thus, the following reasons for the relationship between BNP levels and the severity of ESLD were suggested. ESLD leads to hyperdynamic syndrome in the cardiovascular system, which is a pathophysiology of ESLD 41, 42. It is characterized by increased heart rate and cardiac output and decreased systemic vascular resistance with normal or low blood arterial pressure 43. Increased cardiac performance to maintain appropriate systemic circulation promotes heart wall injury and myocardiocyte stretching, which release BNP into the circulation 44. Therefore, patients with more advanced cirrhosis would have higher levels of BNP in the circulation. In our study, there was also no significant difference in the preoperative EF and diastolic dysfunction because BNP levels could be increased even in the early stage of cardiomyopathy as in asymptomatic patients with cirrhotic cardiomyopathy or silent heart failure 45. The BNP levels in the perioperative period and on postoperative day 1 were higher in the non-survival group than in the survival group and each BNP levels had a significant predictive ability for 1-month mortality after LT in our study. However, cardiac function, intravascular volume status and volume responsiveness such as EF, CVP, SVV, administered fluids, transfused PRBCs, and the numbers of patients with ascites > 1L showed no significant difference. With respect to cardiac function, the proportion of patients with systolic (EF < 50%) and diastolic dysfunction and pulmonary hypertension was higher in the non-survival group than in the survival group although it was not significantly different between the two groups. Thus, asymptomatic cirrhotic cardiomyopathy was associated with elevated BNP levels in patients with ESLD.

The BNP levels at the four time points were significantly different between the survival and non-survival groups in this study (Table 3; *P* < 0.001). All of them had significant predictive abilities for 1-month mortality after LT. We investigated a better predictive model by calculating the BNPs at different time points, even if BNP levels at each individual time point had excellent diagnostic accuracy (Table 4; all of AUROCs > 0.85). We investigated a better predictive model by combining the values at each BNP time point. From the result of conditional logistic regression, the cBNP had an outstanding diagnostic accuracy compared to the BNP levels at single time points, the MELD score, or the MELD-Na score (cBNP AUROC = 0.976). The BNP levels at T2 and T4 were selected, and the BNP levels at T1 and T3 were excluded from the cBNP model by multivariate analysis. We assumed that the reason for the multivariate analysis results was the characteristics of each surgical period. The hemodynamic characteristics at T2 and T4 had more instability than those at the T1 and T3 points. T1 in the pre-anhepatic phase was when the dissection of the recipient's liver was performed. Thus, the period was accompanied by a rapid blood loss and fluid shift due to varices and adhesion in the abdomen and ascitic decompression. T3 in neohepatic phase was performed the reperfusion of the preserved liver. Thus, the period had a massive release of cold, hyperkalemic acidotic fluid into the recipient's circulation 46. Therefore, we assumed that the BNP levels at T2 and T4 were selected by multivariate analysis because those at T1 and T3 would be inappropriate due to hemodynamic instability in the systemic circulation.

The NRI and IDI were calculated to identify diagnostic improvement in using cBNP as a new predictive model compared to the MELD scores. The use of cBNP improved the predictive accuracy of mortality within 1-month after LT 30.1% compared to the MELD score. The IDI also confirmed 0.5% improved diagnostic accuracy of the cBNP (the NRI and IDI between the cBNP and MELD scores; 30.1% and 0.5%; *P* = 0.004, and 0.035, respectively).

We determined the optimal cutoff for the cBNP, BNPs, MELD scores, MELD-Na scores with the best predictive accuracy for mortality within 1-month after LT using AUROC analysis. Using a cBNP cutoff of T2 BNP levels of > 137 pg/mL, and T4 BNP levels of > 187 pg/mL, the sensitivity and specificity of cBNP were as high as 100% and 91.9%, respectively, although the AUROC of cBNP was not significantly different from the AUROC of the BNP levels at single time points except for the T1 BNP levels. Only BNP levels at T1, the MELD score, and the MELD-Na score had significantly different AUROCs. BNP levels at postoperative day 1 (T4) were the second-best predictor and showed sensitivity and specificity as high as 100% and 94.2%, respectively, using a cutoff of 155. The difference in diagnostic accuracy between the cBNP and BNP levels at T4 was 0.013 and the sensitivity and specificity were 100% and > 90%, respectively, both for cBNP and BNP levels at T4. Thus, cBNP can be especially useful to clinicians in predicting whether a patient undergoing LT will have a high risk for mortality after transplant (Table 4). BNP levels at T4 are also useful to clinicians, if BNP levels at two time points are not available.

There were several limitations to the interpretation of the results in this study. First, the clinical heterogeneity of the causes of mortality was not adjusted in the study. Second, we were unable to identify pathogenic mechanisms linking cardiac dysfunction, serum BNP levels, and the severity of liver disease. Only the preoperative EF and systolic and diastolic dysfunction by echocardiography were adjusted for cardiac dysfunction. There were limited variables for cardiac evaluation due to the retrospective nature of the study. Third, the use of diuretics and beta-blockers in patients with advanced liver diseases and hypertension have biased our results by underestimating cardiac alterations in patients with advanced liver disease. Fourth, the majority of ESLD in the study population was caused by hepatitis B virus infection, which is a predominant cause of ESLD in Asia. Finally, there are no established guidelines for treating patients with elevated BNP levels, and it is hypothesized that the severity of advanced liver diseases is correlated with elevated levels of serum BNP.

In conclusion, our findings support the outstanding prognostic power of cBNP and its usefulness as a predictive model of patient mortality within one month of LT. The diagnostic accuracy of cBNP was increased by as much as 30.1% compared to the MELD scores based on the results of the NRI, IDI, and AUROCs. We evaluated the prognostic ability of BNP levels in patients undergoing LT and found that it could provide helpful information to transplant clinicians for predicting patient mortality and providing the best medical treatment. These putative roles require further investigation in a larger, prospective randomized control study, as well as the external validation of our suggested cBNP model. Further studies should be conducted to explore whether potential interventions such as the use of diuretics and beta-blockers could reduce BNP levels and further improve patient prognosis.

## Authorship

- **Conceptualization:** Hyun Sik Chung, Yun Sung Jo
- **Data curation:** Hyun Sik Chung, AMi Woo
- **Formal analysis:** Hyun Sik Chung, MinSuk Chae, Sang Hyun Hong, Chul Soo Park
- **Investigation:** Hyun Sik Chung, AMi Woo
- **Methodology:** Hyun Sik Chung, Jong Ho Choi
- **Project administration:** MinSuk Chae, Sang Hyun Hong, Chul Soo Park
- **Writing - original draft:** Hyun Sik Chung
- **Writing - review & editing:** Jong Ho Choi, Yun Sung Jo.

## Figures and Tables

**Table 1:** Demographic and preoperative data of the study population

Characteristic	Survival (N=173)	Non-survival (N=14)	*P* value
**Recipient**			
Age (years)	52±9	52±13	0.942
Gender (female/male)	54 (31.2)/119 (68.8)	5 (35.7)/9 (64.3)	0.768
**Causes of ESLD**			
Hepatitis B	70 (40.5)	2 (14.3)	0.025*
Hepatitis C	23 (13.3)	2 (14.3)	
Hepatitis A	5 (2.9)	3 (21.4)	
Alcoholic	22 (12.7)	2 (14.3)	
HCC	31 (17.9)	3 (21.4)	
Others	22 (12.7)	2 (14.3)	
BMI (kg/m^2^)	24.9±4.0	25.3±4.5	0.720
MELD (pts)	18±11	29±14	0.017*
MELD-Na (pts)	17±13	27±17	0.040*
**Serum**			
Sodium (mmol/L)	138.1±6.0	140.2±3.7	0.199
Lactate (mg/dL)	2.6±2.5	5.4±4.5	0.238
bilirubin (total, mg/dL)	8.0±11.2	14.0±15.3	0.172
albumin (mg/dL)	3.1±0.6	2.3±1.0	0.986
AST (U/L)	167.8±608.1	745.9±1555.1	0.190
ALT (U/L)	146.5±552.1	923.0±1819.3	0.135
INR	1.73±0.77	2.29±1.04	0.012*
Creatinine (mg/dL)	1.22±1.10	2.15±1.26	0.003*
C-RP (mg/L)	1.39±2.34	3.14±2.71	0.009*
Ejection fraction (%)	64.4±5.0	62.7±5.1	0.349
Ejection fraction < 50 %	2 (1.2)	1 (7.1)	0.220
Diastolic dysfunction	96 (55.5)	11 (64.7)	0.093
Pulmonary hypertension	9 (5.2)	2 (14.3)	0.120
**Donor**			
Age (years)	35±13	42±12	0.106
Gender (female/male)	62 (35.8)/111 (64.2)	5 (35.7)/9 (64.3)	1.000
Ischemic time (min)	115±73	94±48	0.347
Living / Deceased	134 (77.5)/39 (22.5)	11 (78.9)/3 (21.1)	1.000
GWRW	1.18±0.45	1.39±0.56	0.120
Fatty change of graft (%)	3.5±6.1	4.7±5.1	0.484

Data are presented as mean ± SD, or numbers (%). ESLD, end-stage liver disease; HCC, hepatocellular carcinoma; BMI, body mass index; MELD, model for end-stage liver disease; MELD-Na, sodium conjugated model for end-stage live disease; AST, asparate transaminase; ALT, alanine transaminase; INR, international normalized ratio; GWRW, graft weight to recipient weight.*Statistically significant differences (*P* value of < 0.05).

**Table 2:** Laboratory and hemodynamic data according to the three phases of liver transplantation in the survival and the non-survival groups

Characteristic	Survival (N=173)	Non-survival (N=14)	*P* value
**BNP (pg/mL)**			
T1	87.6 (45.5-171.0)	307.9 (174.2-828.1)	<0.001*
T2	79.0 (40.5-121.3)	400.5 (215.5-445.3)	<0.001*
T3	82.9 (41.6-128.3)	216.2 (189.0-291.0)	<0.001*
T4	106.0 (73.0-127.0)	214.5 (174.7-242.8)	<0.001*
**Creatinine (mg/dL)**			
T1	1.11±0.48	1.56±1.38	0.249
T2	1.28±0.81	1.15±0.33	0.549
T3	1.19±0.65	1.00±0.11	0.255
**Glucose (mg/dL)**			
T1	142±42.0	113±46.0	0.697
T2	154±51.0	140±52.0	0.382
T3	208±51.0	183±49.0	0.167
**Neutrophile-to-Lymphocyte ratio**			
T1	2.90 (1.57-5.92)	3.47 (2.51-11.28)	0.119
T2	7.67 (4.68-12.43)	9.88 (1.93-11.65)	0.606
T3	11.17 (7.31-16.75)	12.99 (1.94-18.29)	0.625
T4	17.42 (10.67-24.37)	14.30 (8.29-19.97)	0.223
**pH**			
T1	7.40±0.07	7.33±0.09	<0.001*
T2	7.28±0.10	7.24±0.12	0.158
T3	7.30±0.08	7.28±0.11	0.263
**Lactate (mg/dL)**			
T1	2.60±3.50	3.80±4.16	0.225
T2	5.82±3.05	6.13±3.78	0.745
T3	5.72±3.39	5.55±3.39	0.861
**Transfusion PRBCs (unit)**			
T1	3.8±4.4	6.9±7.2	0.144
T2	2.8±4.0	2.8±2.3	0.959
T3	3.1±3.4	3.8±3.8	0.525
All periods	9.9±9.1	13.6±7.1	0.083
**Administered fluids**			
Crystalloids (L)	6.8±3.4	6.4±2.7	0.678
Colloids (mL)	805±476	746±552	0.664
**Ascites >1L**	79 (45.7)	8 (57.1)	0.408
***Hemodynamics***			
**HR (beat/min)**			
T1	84±16	91±17	0.125
T2	94±18	95±23	0.953
T3	95±14	95±20	0.937
**mABP (mmHg)**			
T1	76±14	81±14	0.583
T2	73±14	79±12	0.055
T3	76±47	74±12	0.833
**CVP (mmHg)**			
T1	10±4	11±5	0.374
T2	9±4	10±4	0.393
T3	11±4	14±4	0.026*
**SVV (%)**			
T1	7.3±3.9	5.3±2.1	0.253
T2	9.2±8.0	7.8±4.7	0.595
T3	6.7±4.1	6.9±4.5	0.854
**CI (L/min/m^2^)**			
T1	4.0±1.1	4.1±0.7	0.349
T2	3.8±1.2	4.1±1.2	0.644
T3	4.8±1.3	4.7±1.3	0.697
**SVRI (dynes-sec/cm^-5^/m^2^)**			
T1	1338±453	1334±386	0.891
T2	1404±545	1505±859	0.626
T3	1077±417	1054±445	0.881
**Operation time (hr)**	8.6±1.5	8.4±2.1	0.569

Data are presented as mean ± SD or numbers (%). T1, 60 mins into pre-anhepatic phase; T2, 30 mins into anhepatic phase; T3, 30 mins after reperfusion of the grafted liver (neohepatic phase); T4, postoperative day 1. BNP, B-type natriuretic peptide; PRBC, packed red blood cell; HR, heart rate; mABP, mean arterial blood pressure; CVP, central venous pressure; SVV, stroke volume variation; CI, cardiac index; SVRI, systemic vascular resistance index.

**Table 3:** Multivariate analysis including the odds ratio of the BNP and Cr at time points between the survival and the non-survival groups

	Survival (N=173)	Non-survival (N=14)	*P* value	Odds ratio (95% CI)	*P* value
**BNP (pg/mL)**					
T1	87.6 (45.5-171.0)	307.9 (174.2-828.1)	<0.001*		
T2	79.0 (40.5-121.3)	400.5 (215.5-445.3)	<0.001*	1.010 (1.000-1.020)	0.003
T3	82.9 (41.6-128.3)	216.2 (189.0-291.0)	<0.001*		
T4	106.0 (73.0-127.0)	214.5 (174.7-242.8)	<0.001*	1.011 (1.001-1.026)	0.010
**LN BNP**					
T1	4.44 ±1.00	5.88±0.78	<0.001*		
T2	4.25±0.82	5.81±0.44	<0.001*	12.9 (2.349-70.963)	0.003
T3	4.28±0.88	5.46±0.29	<0.001*		
T4	4.55±0.51	5.36±0.21	<0.001*	12.0 (1.006-142.759)	0.049

LN BNP; log-transformation of the BNP levels at each time point; BNP, B-natriuretic peptide; CI, confidence interval. T1, 60 mins into pre-anhepatic phase; T2, 30 mins into anhepatic phase; T3, 30 mins after reperfusion of the grafted liver (neohepatic phase); T4, postoperative day 1. The analysis was adjusted for international normalized ratio, creatinine, C-reactive protein, and ejection fraction.

**Table 4:** Comparison of the predictive values, sensitivity, specificity, diagnostic accuracy, and differences in AUROCs of the cBNP, BNP, Cr, MELD scores, and MELD-Na scores for predicting 1-month mortality after liver transplantation

Prognostic test	Threshold	Sensitivity	Specificity	PPV	NPV	Diagnostic accuracy	ΔAUROC	95% CI	*P* value^a^
***cBNP score***									
**BNP (pg/mL)**									
T2	> 137	100.0	91.9	50.0	100.0	0.976	Ref.	0.94-0.99	Ref.
T4	> 187								
**MELD (pts)**	> 31	57.1	85.9	25.0	96.1	0.714	0.262	0.64-0.78	0.001*
**MELD-Na (pts)**	>29	57.1	82.9	21.6	95.9	0.690	0.286	0.62-0.76	<0.001*
**BNP (pg/mL)**									
T1	> 124	100.0	64.7	18.7	100.0	0.865	0.111	0.81-0.91	0.021*
T2	> 175	92.9	89.6	41.9	99.4	0.962	0.015	0.92-0.99	0.545
T3	> 150	100.0	80.3	29.2	100.0	0.913	0.063	0.86-0.95	0.210
T4	> 155	100.0	94.2	58.3	100.0	0.963	0.013	0.93-0.99	0.723

BNP, B-type natriuretic peptide; cBNP, combined BNP; MELD, model for end-stage liver disease; MELD-Na, sodium conjugated model for end-stage live disease; PPV, positive predictive value; NPV, negative predictive value; AUROC, area under the receiver operating characteristic curve; ΔAUROC, difference in AUROCs. T1, 60 mins into pre-anhepatic phase; T2, 30 mins into anhepatic phase; T3, 30 mins after reperfusion of the grafted liver (neohepatic phase); T4, post-operative day 1. ^a^*P* value calculated for the comparison of cBNP vs. the other models. *Statistically significant differences (*P* value of < 0.05).

**Table 5:** Reclassification of predicted of 1-month mortality risk after LT between the cBNP and MELD scoring systems

		Model using cBNP					Reclassified		Net correctly
Model using MELD score	***Survival (non-cases, n = 173)***	***< 5%***	***5-15%***	***15-20%***	***≥ 20%***	***Total***	***Increased risk***	***Decreased risk***	***Reclassified (%)***
	< 5%	94 (54.3)	4 (2.3)	0 (0)	0 (0)	98			
	5%-15%	**46 (26.6)**	5 (2.9)	1 (0.6)	5 (2.9)	57	11	63	30.1
	15%-20%	**6 (3.5)**	**3 (1.7)**	0 (0)	1 (0.6)	38			
	≥ 20%	**7 (4.0)**	**1 (0.6)**	**0 (0)**	0 (0)	8			
	***Non-survival (cases, n = 14)***	***< 5%***	***5-15%***	***15-20%***	***≥ 20%***	***Total***			
	< 5%	0 (0)	**1 (7.1)**	**0 (0)**	**3 (21.4)**	4			
	5%-15%	0 (0)	1 (7.1)	**0 (0)**	**3 (21.4)**	4	8	2	42.9
	15%-20%	0 (0)	0 (0)	0 (0)	**1 (7.1)**	1			
	≥ 20%	0 (0)	1 (7.1)	1 (7.1)	3 (21.4)	14			
	**Net reclassification improvement (95 % CI, P value)**				0.301 (0.224-0.396, 0.004)				
	**Integrated discrimination improvement (95 % CI, P value)**				0.005 (0.027-0.076, 0.035)				

## Abbreviations

AUROC: area under the receiver operating characteristic curve
BMI: body mass index
BNP: B-type natriuretic peptide
cBNP: combined B-type natriuretic peptide
ESLD: end-stage liver disease
EF: ejection fraction
LT: liver transplantation
MELD: model for end-stage liver disease
MELD-Na: incorporated sodium into MELD
GWRW: graft weight to recipient weight
